# Identification and functional analysis of a novel *SMARCC2* splicing variant in a family with syndromic neurodevelopmental disorder

**DOI:** 10.1186/s13023-024-03510-5

**Published:** 2025-02-03

**Authors:** Ming Li, Jingqi Lin, Hongjun Fei, Jinyu Liu, Yiyao Chen, Xu Han, Yanlin Wang, Jian Wang, Renyi Hua, Shuyuan Li, Niu Li

**Affiliations:** 1 The International Peace Maternity and Child Health Hospital, Shanghai Jiao Tong University School of Medicine, Shanghai, 200030 China; 2 Shanghai Key Laboratory of Embryo Original Diseases, Shanghai, 200030 China; 3 Institute of Birth Defects and Rare Diseases, Shanghai Jiao Tong University School of Medicine, Shanghai, 200030 China; 4 Faculty of Medical Laboratory Science, College of Health Science and Technology, Shanghai Jiao Tong University School of Medicine, Shanghai, 200025 China

**Keywords:** Syndromic neurodevelopmental disorder, *SMARCC2*, Novel splicing variant, cDNA sequencing, Crystal structure analysis

## Abstract

**Background:**

To determine the pathogenicity of a novel splicing variant in the *SMARCC2* gene identified from a pair of adult male monozygotic twins with neurodevelopmental disorder, and to investigate the genotype-phenotype characteristics associated with *SMARCC2* variants.

**Methods:**

Whole-exome sequencing (WES) was conducted on the proband, and candidate variants were validated using Sanger sequencing within the family. The effect of the identified splicing variant on *SMARCC2* mRNA processing was analyzed using reverse transcription PCR (RT-PCR) and TA-clone sequencing using samples derived from the proband. The clinical features of the twins were collected and compared with the previously reported patients.

**Results:**

The twin adult males displayed comparable phenotypes, characterized by moderate developmental delay, intellectual and language delays, dense hair, craniofacial anomalies, scoliosis, cryptorchidism, hypotonia, behavioral abnormalities, allergic purpura and eczema, and drug allergies. WES unveiled a previously unreported heterozygous splice variant of the *SMARCC2* gene (NM_003075.3: c.1496 + 1G > T). Sanger sequencing confirmed that the variant was *de novo* in both patients. TA-clone sequencing of the RT-PCR fragments showed that the canonical splicing variant resulted in two distinct aberrant splicing events in *SMARCC2* mRNA. Specifically, approximately 80% of the mutant clones resulted from the in-frame insertion of 126 bases in intron 16, while the remaining 20% showed an in-frame deletion of exon 16 (c.1383_1496del). Crystal structure analysis showed that both in-frame alterations hindered the proper formation of the alpha helix structure within the SMARCC2 protein. An analysis of genotype-phenotype correlations indicated that our patients displayed neurological phenotypes of greater severity than those observed in patients with truncating variants, instead aligning more closely with the characteristics of the missense/in-frame variant group.

**Conclusion:**

We identified and reported a pair of twins suffering from syndromic neurodevelopmental disorders caused by a novel splicing variant of *SMARCC2*. Our findings further reinforce the notion that individuals harboring missense/in-frame variants in SMARCC2 are prone to experiencing more severe neurological phenotypes.

## Introduction

As one of the most important chromatin-remodeling complexes in mammalian cells, the BRG1-associated factor (BAF) complex (also known as the SWI/SNF complex) plays a critical role in regulating target gene expression by modulating nucleosomes and altering chromosomal conformation and accessibility [1, 2]. The BAF complex is primarily composed of *ARID1A*, *ARID1B*, *SMARCA4*, *SMARCB1*, *SMARCE1*, and *SMARCC2*, and heterozygous variant in either of them can lead to several distinct neurodevelopmental disorders (NDDs) with overlapping phenotypes, termed BAFopathies. The most well characterized BAFopathies include Coffin-Siris syndrome (CSS, #MIM 135900) and Nicolaides-Baraitser syndrome (NCBRS, #MIM 601358) [3–6]. CSS was first reported by Coffin and Siris in 1970, and to date, at least 12 subtypes have been included in the Online Mendelian Inheritance in Man (OMIM) database (https://omim.org/). Among these, *ARID1B* is the most frequently mutated gene [7]. Generally, CSS has broad and variable clinical features, mainly involving a range of developmental delay (DD), intellectual disability (ID), speech delay, facial dysmorphic features, hypotonia, hypertrichosis, hypoplasia of the fifth fingernail or distal phalanx, and sparse scalp hair [8–11]. Currently, there are no formal clinical diagnostic guidelines or consensus for CSS; therefore, the recognition and diagnosis of patients with CSS mainly rely on genetic testing.

The *SMARCC2* gene, which is located on chromosome 12q13 and encodes the protein BAF170 composed of 1, 214 amino acids, is essential for the development of glial radial cells that control the cortical architecture [12]. Although previous sporadic cases from large NDD cohorts [13, 14] and studies in mice have suggested that *SMARCC2* may be a disease-causing gene related to neurological defects [12, 15], it was not until 2019 that an association between *SMARCC2* variant and specific diseases was established [6]. In the OMIM database, *SMARCC2*-associated phenotype was defined as CSS8 (#MIM 618362) mainly based on the detail clinical description of the first 15 patients [6]. Subsequently, new *SMARCC2* mutant patients were discovered. Bosch et al. recently summarized the clinical characteristics of 65 patients and found that the similarity between the *SMARCC2*-associated phenotype and CSS or NCBRS was limited, which verifies the strong clinical heterogeneity and suggests that the naming of this disease requires further discussion [16]. Most of the reported individuals were pediatric patients, but there was also a certain proportion of adult patients, indicating that the *SMARCC2* variant may not have a significant impact on lifespan. This also means that most patients continuously demand clinical monitoring and evaluation, such as fertility assessment, during the follow-up period. Therefore, it is important to comprehensively understand the natural history of *SMARCC2*-related NDD by collecting and describing the clinical characteristics of patients in various age groups.

In this study, we present a pair of adult male twins suspected of having syndromic NDD. Genetic sequencing identified a previously unreported splicing variant of *SMARCC2*, which may explain the condition of the family. The effect of the splicing variant on *SMARCC2* was determined using TA-clone sequencing of peripheral blood samples from the proband. This study represents one of the few reported cases in adults, not only expanding the mutant spectrum of *SMARCC2* but also enhancing our understanding of the natural history of *SMARCC2*-related NDD.

## Materials and methods

### Patients

A married male who has been diagnosed with sexual dysfunction was referred to our hospital for a consultation on fertility strategies. The proband and his elder twin brother came from a Chinese family in which the parents were healthy and nonconsanguineous. Preliminary examination at the outpatient clinic suggested that both twins had syndromic neurodevelopmental disorders and were recommended for further genetic testing.

### WES and data analysis

Proband-only whole-exome sequencing (WES) was performed as previously described [17]. Briefly, genomic DNA was extracted from peripheral blood using a QIAamp DNA Blood Mini Kit (Qiagen, Hilden, Germany). The sequencing library was prepared using KAPA HyperExome V3 Probes (Roche, Basel, Switzerland) and then sequenced on the MGISEQ-2000 platform (BGI, Shenzhen, China). Quality control criteria for sequencing data included: (1) average sequencing depth of the target regions ≥ 180X; (2) with > 95% of target regions having an average depth > 20X. Sequencing reads were aligned to the UCSC hg19 human reference genome using the Burrows-Wheeler Alignment (BWA) tool and duplicated reads were removed. Single-nucleotide variants and small indels were identified using GATK. All variants were saved in VCF format and uploaded to the TGex (Translational Genomic Expert) system. Exome-level copy number variations (CNV) were analyzed using ExomeDepth software.

### Sanger sequencing validation of the identified *SMARCC2* variant

Genomic DNA was extracted from peripheral blood samples of the elderly brother and parents. Primers were designed using Primer 5.0, based on the human *SMARCC2* gene sequence (NM_003075.3) and synthesized by BGI (Shanghai, China). Primers were designed for exon 16 (forward: 5′- AGCTACCTGGCCTATCGAAA − 3′ and reverse: 5′- AGGCTGGTCTCGAACTTCTG − 3′). The targeted exons and exon-intron boundaries were amplified using polymerase chain reaction with the following conditions: pre-denaturation at 95 °C for 5 min, denaturation at 94 °C for 30 s, annealing at 56 °C for 30 s, extension at 72 °C for 30 s, cycling for 35 cycles; final extension at 72 °C for 10 min and an indefinite 4 °C hold. Sanger sequencing was performed with both forward and reverse primers using an ABI3500 sequencer (Applied Biosystems, Foster City, CA, USA). Data were analyzed using the CodonCode Aligner software (CodonCode Corporation, Centerville, MA, USA).

### Reverse transcription PCR (RT-PCR) and TA-clone sequencing

Peripheral blood mononuclear cells (PBMCs) were isolated from the proband and his healthy father using Ficoll-Paque plus (GE, Fairfield, CT), and total RNA was extracted using the QIAamp RNA Blood Mini Kit (Qiagen). cDNA was obtained using the Hifair AdvanceFast 1st Strand cDNA Synthesis SuperMix kit (Yeasen, Shanghai, China). The following primers were used to amplify a 654-bp fragment of the wild-type *SMARCC2* cDNA: forward, 5′-GATGAGGATGAGAACAGTAC-3′; reverse, 5′-CTCCAGGAGAAGCAGGGTTT-3′. The forward and reverse primer sequences were located in exons 14 and 19, respectively. The PCR products were visualized using electrophoresis on a 1% agarose gel after staining with ethidium bromide. *GAPDH* was used as an internal control to assess RNA quality, and the following primers were used to amplify the *GAPDH* fragment: forward, 5′- GAGATCCTTCCAAAATCAAGT-3′ and reverse, 5′-GTGGAGGAGTGGGTGTCG-3′. The wild-type (WT) and mutant *SMARCC2* fragments were cloned into the PMD-18-T vector (TaKaRa) and transformed into the Escherichia coli TOP10 reference strain. The clones were selected using a blue-white spot screening plate. Clone sequencing was performed to identify PCR products.

### Three-dimensional structure analysis of the SMARCC2 mutant protein

The crystal structure of the wild-type SMARCC2 was obtained from the Protein Data Bank (https://www.rcsb.org/structure/6KAG) and was generated using Pymol software (v.1.8.4.0, Schrödinger Inc., New York, NY, USA). The mutated SMARCC2 was modeled using the Swiss model software (https://swissmodel.expasy.org/) after replacing amino acids Ile461 to Arg499 with methionine (p.I461-R499delinsMet) or inserting 42 novel amino acids between Met498 and Arg499 (p.M498-R499insSWVLWLRGYMRMPAYGCVLATVEMPARNTEQTEKSTSRE), and the three-dimensional structure of the mutated SMARCC2 proteins were then constructed by PyMOL, respectively.

## Results

### Patient description

**Fig. 1:**
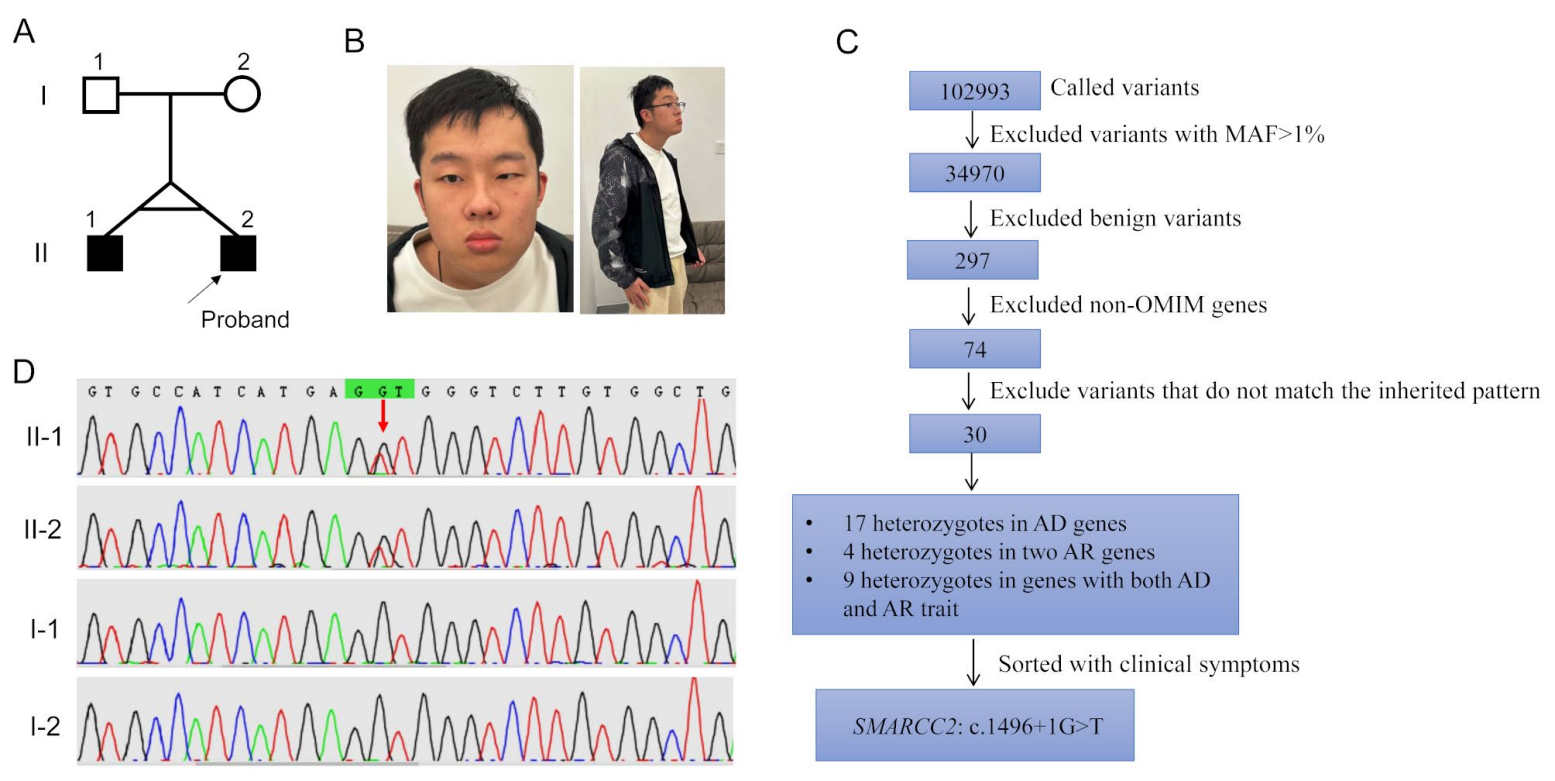
Identification and description of the patient along with the *SMARCC2* variant. (**A**) Pedigree of the family. Males are indicated by squares and females are indicated by circles. Generations are labeled using Roman numerals (I, II). Solid symbols indicate affected individuals. (**B**) Photograph to highlight facial anomalies, including small left eye, epicanthus, low-set ears, wide nose and thick lower lip, and scoliosis in the proband. (**C**) The data analysis algorithm used for filtering all single nucleotide variants identified using whole exome sequencing, with the number of remaining variants after each filtering step. On filtering and prioritization, heterozygous variant of the *SMARCC2* gene was identified as the top candidate. AD, autosomal dominant; AR, autosomal recessive; MAF, minor allele frequency; OMIM: Online Mendelian Inheritance in Man. (**D**) Sanger sequencing to confirm the c.1496 + 1G > T variant in the *SMARCC2* gene in this family

The proband (II-2) was a 24-year-old married male with an older monozygotic twin brother (Fig. 1A). The clinical features of the twins are summarized in Table 1. The twins did not exhibit any obvious abnormalities at birth. At three years of age, the parents noticed that they had moderate DD/ID and language delay. The twins were able to engage in simple language communication but could not speak long or complex sentences. They had basic self-care abilities but could not perform fine motor skills. The proband had a history of epilepsy and two onsets at the ages of 3 and 18 years. He had undergone cryptorchidism surgery during childhood and was diagnosed with strabismus at 13 years of age. Craniofacial anomalies, including a prominent forehead, small left eye, epicanthus, low-set ears, wide nose, and thick lower lip, were observed in the proband (Fig. 1B).

**Table 1:** Clinical characteristics of the twins with *SMARCC2* variant

	Proband (II-2)	Elder brother (II-1)	Reported patients (*n* = 65)
**Basic information**			
Sex	Male	Male	24 female, 37 male, 4 unknown
Age at last investigation	24	24	≥ 18year (11), < 18 (47), fetus (1), died in perinatal period(1), 5 unknown
Variant	c.1496 + 1G > T	c.1496 + 1G > T	29 truncating, 26 missense/ in-frame
Segregation/Inheritance	De novo	De novo	De novo (34/54)
**Body and craniofacial deformities**			
Sparse hair	No	No	9/57
Dense hair	Yes	Yes	Not reported
Abnormality of the eye	Yes (strabismus, small left eye)	Yes (strabismus)	14/48
Abnormality of the outer ear	Yes (low set ears)	Yes (low set ears)	20/44
Epicanthus	Yes	Yes	Not reported
wide nose	Yes	Yes	13/51
thick lower lip	Yes	Yes	16/53
Scoliosis	Yes	Yes	15/54
Generalized abnormality of skin	Yes (allergic purpura and eczema)	Yes (allergic purpura and eczema)	9/58
**Neurological development**			
Intellectual disability	Yes, moderate	Yes, moderate	47/55
Speech abilities	Yes (Delayed language development)	Yes (Delayed language development)	47/57
Behavioral abnormalities	Yes (irritability, mysophobia, negative manifestations)	Yes (irritability, mysophobia, negative manifestations)	32/53
Hypotonia	Yes	Yes	40/58
Seizures	Yes(Long-term drug control, no abnormality was found in EEG and skull MRI)	Yes(Long-term drug control), No abnormality was found in EEG and skull CT	15/54
Hernia	Yes (onset of 8–9 age)	No	7/55
**Other anomalies**			
Abnormality of the genitourinary system	Yes (enlarged prostate, erectile dysfunction, cryptorchidism)	Yes (no details)	8/37
Drug allergy	penicillin, aminopyrine allergies, and ketamine	penicillin, aminopyrine allergies, and ketamine	Not reported
Erectile dysfunction	Yes	Yes	Not reported

Both brothers had scoliosis, allergic purpura, eczema (12–13 years old), a similar history of drug allergies (penicillin, aminopyrine allergies, and ketamine), hypotonia, behavioral abnormalities (irritability, mysophobia, negative manifestations), sexual dysfunction, and dense hair. No abnormalities were found in height, weight, electroencephalogram, skull magnetic resonance imaging, or chromosome karyotype of the twins.

The parents realized that the twins might have suffered from a congenital disease. The family actively treated each symptom during the growth process. However, owing to the complexity of the phenotype, they believed that it may be difficult to develop a fundamental treatment method, thus the twins had not undergone genetic testing before. Due to the need for reproduction, the proband underwent a detailed sexual development examination and was diagnosed with an enlarged prostate and erectile dysfunction. Bilateral testicular development was normal, with 16 and 18 mL on the left and right sides, respectively. Sperm examination revealed no significant damage to integrity, maturity, or vitality.

### Identification of the *de novo* splicing variant in the *SMARCC2* gene

Candidate variants were initially excluded based on the following criteria: (1) common variants with allele frequencies greater than 1% in the gnomAD database (https://gnomad.broadinstitute.org/); (2) benign variants predicted using various in silico tools, mainly including REVEL [18] and Splice AI [19]; (3) variants in non-OMIM genes; and (4) variants that do not meet the diagnostic criteria according to the inheritance pattern (i.e., heterozygotes in recessive genes). Finally, WES identified a heterozygous splicing variant (c.1496 + 1G > T) in the *SMARCC2* gene, which has not been reported previously and has not been included in any public database (Fig. 1C). Additionally, Sanger sequencing confirmed that both the proband and his elder brother harbored the c.1496 + 1G > T variant, while the allele of their parents was wild-type, suggesting *de novo* status of the variant in the twins (Fig. 1D).

### The c.1496 + 1G > T variant causes aberrant splicing of *SMARCC2* mRNA

**Fig. 2:**
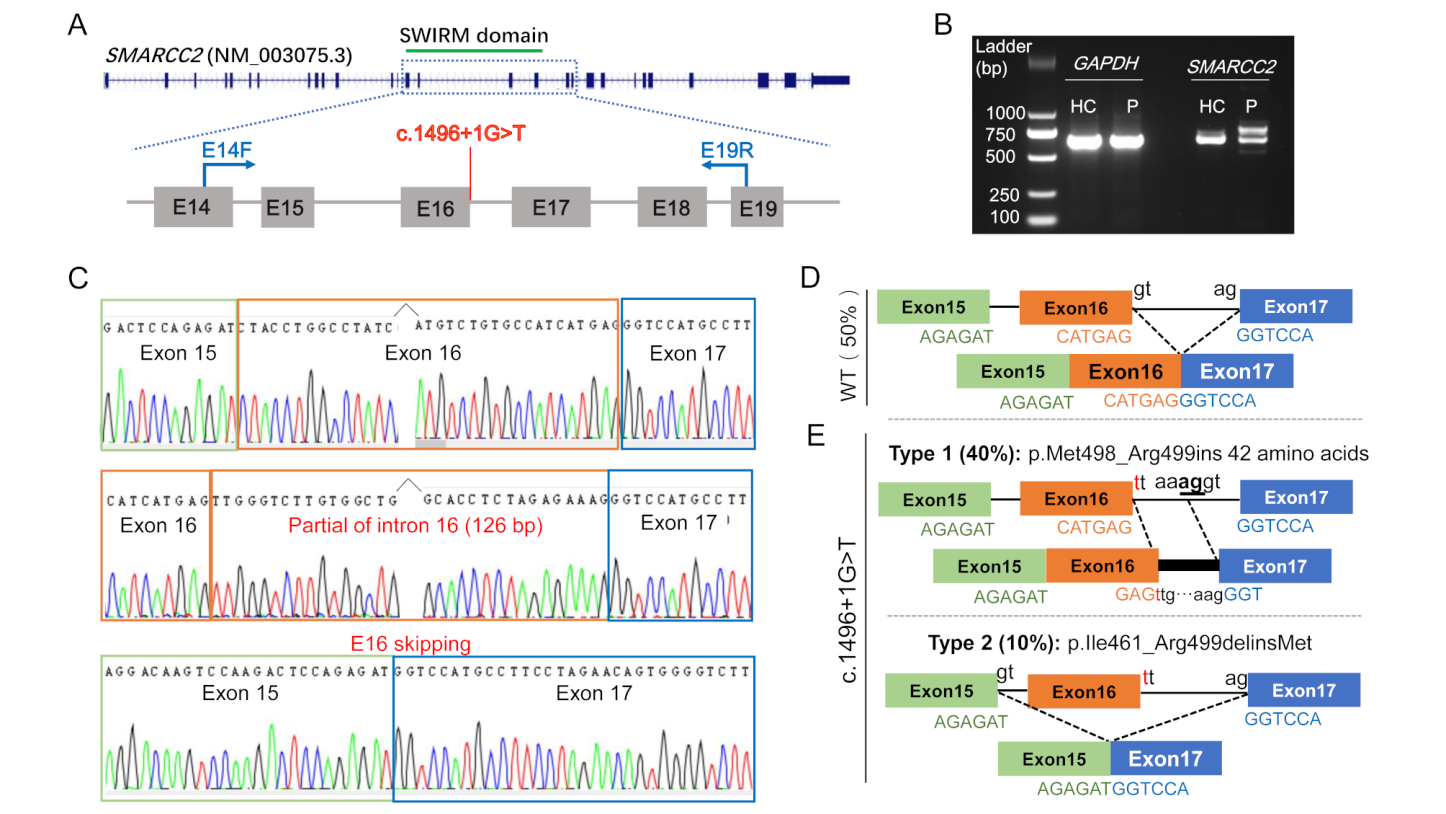
Electrophoresis and TA-clone assays for the c.1496 + 1G > T variant. (**A**) The schematic diagram to show the position of the c.1496 + 1G > T variant and the primers location that were used to amplify the cDNA fragments of the *SMARCC2*. (**B**) Electrophoresis of the RT-PCR products to show the aberrant splicing caused by the c.1496 + 1G > T variant. HC, healthy control (I-1); P, proband (II-2). (**C**) TA-clone assays showed that half of the sequenced clones had abnormal splicing events, including 80% harboring 126 bases retention in intron 16 (c.1496_1497insttgggtcttgtggctgaggggaagggggtacatgaggatgcctgcctatggatgtgttttggcaacagtagagatgccagcaaggaacacagaggagcagacagaaaaaagcacctctagagaaag, p.Met498_Arg499insSWVLWLRGRGYMRMPAYGCVLATVEMPARNTEEQTEKSTSRE) and 20% resulting in exon 16 skipping (c.1383_1496del, p.Ile461_Arg499delinsMet). (**D**) Schematic representation of the wild-type (WT) mRNA transcript. In the WT, splicing occurred at the authentic splice site and the cDNA can be spliced normally. (**E**) Schematic representation of the mutant mRNA transcripts caused by the c.1496 + 1G > T variant

The c.1496 + 1G > T variant locates in intron 16 of *SMARCC2*, where the adjacent exon region encodes the key SWIRM domain (Fig. 2A). The SpliceAI tool predicted that the c.1496 + 1G > T variant was highly likely to cause loss of the donor splice site (score = 1.0); however, there was also a moderate possibility of obtaining a new donor splice site (score = 0.52). To assess the effects of the c.1496 + 1G > T variant on splicing, *SMARCC2* cDNA fragments were obtained from the proband and his healthy father. Surprisingly, only two bands were detected in the normal control sample (Fig. 2B). TA-clone sequencing showed that 75% of the clones were 654-bp products, and the other 25% were novel transcripts with an extra 93 bp from intron 18 (ggccgccaggttgatgctgataccaaggctgggcgaaagggcaaagagctggatgacctggtgccagagacggctaagggcaagccagagctg). Compared to the control sample, the mutant *SMARCC2* generated four bands of increased size (Fig. 2B). TA clone sequencing showed that both the wild-type and novel transcripts were present in the patient, accounting for 50% of the total clones (Fig. 2C and D). For the remaining half of the clones, 80% had 126 bases retention of the initial region of intron 16, resulting in an in-frame insertion of 42 amino acids into the SMARCC2 protein (p.Met498_Arg499insSWVLWLRGRGYMRMPAYGCVLATVEMPARNTEEQTEKSTSRE). The remaining 20% resulted from the skipping of exon 16 (c.1383_1496del, p.Ile461_Arg499delinsMet) (Fig. 2C and E). These results suggest that the c.1496 + 1G > T variant not only causes loss of the donor splice site, presenting as exon 16 skipping, but also leads to the activation of the hidden splicing site in intron 16.

### The c.1496 + 1G > T variant impairs the correct formation of SMARCC2 protein structure

**Fig. 3:**
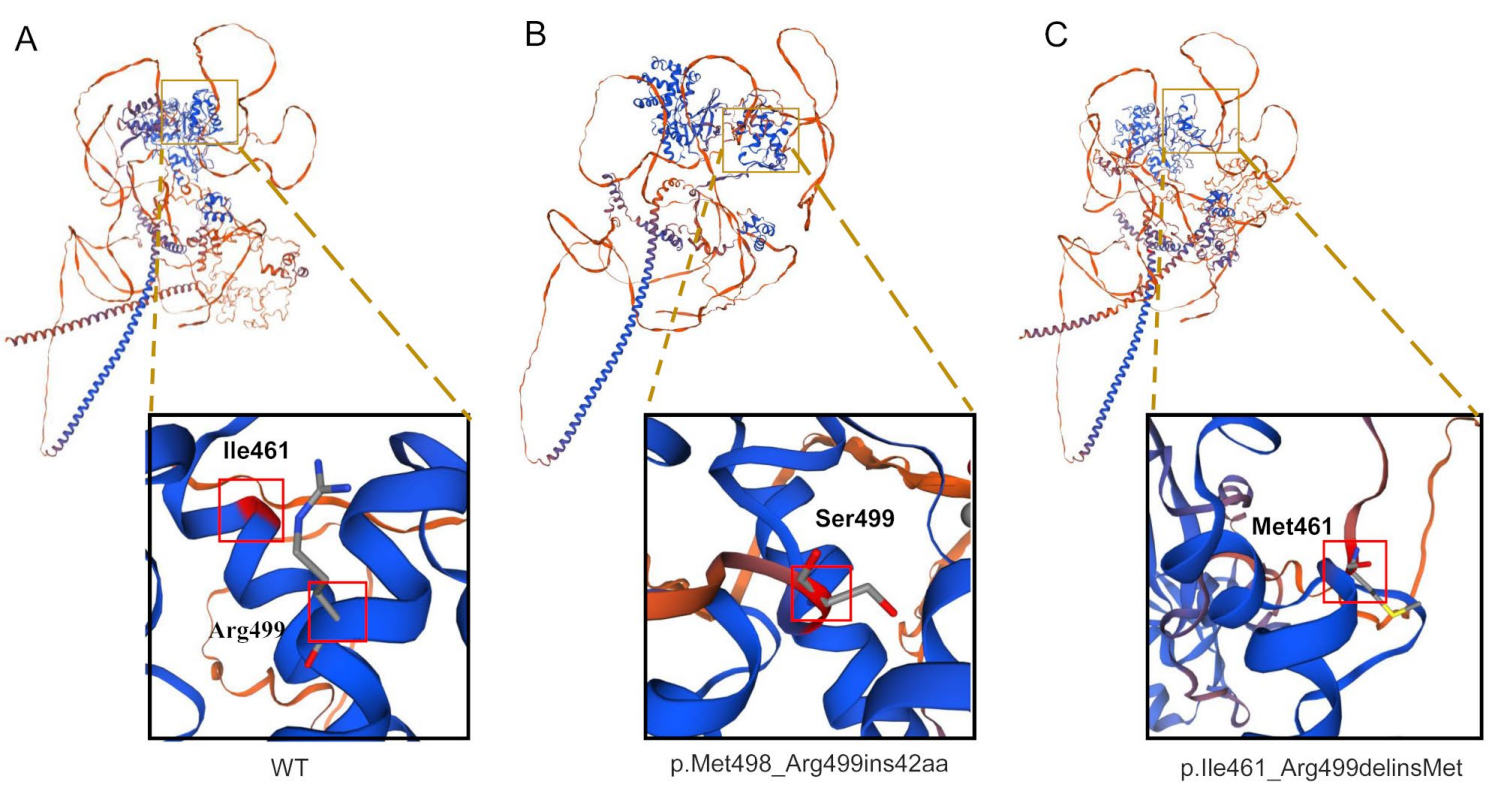
Three-dimensional molecular structure modeling of the SMARCC2 protein. (**A**) The crystal structure of the wild-type (WT) SMARCC2 shows that the Ile461 to Arg499 residues locate in the region composed of α-helices. (**B**, **C**) Both the p.Met498_Arg499ins42aa and p.Ile461_Arg499delinsMet variants result in the disruption of α-helices

To further analyze the impact of amino acid changes caused by the c.1496 + 1G > T variant on protein structure, we model the two mutants in the SMARCC2 wild-type crystal structure. The Ile461 to Arg499 amino acid residues form multiple alpha helix structures (Fig. 3A); however, insertion of 42 amino acids between Met498 and Arg499 or Ile461_Arg499delinsMet can cause defects in the formation of such important structures (Fig. 3B and C). It is well-established that the region spanning from Ile461 to Arg499 is situated within the SWIRM domain, a conserved alpha-helical structure that holds a crucial function in regulating gene expression and facilitating protein-protein interactions [1, 20]. Since both deletions and insertions can disrupt the formation of alpha helices within the SWIRM domain, it is postulated that these mutations will result in protein folding defects, ultimately leading to a reduction in the stability of SMARCC2. According to the ACMG guidelines [21], we conducted a pathogenicity analysis using the evidence clauses PS2, PM2-supporting, PP3, and PP4. Based on the results of the protein structure analysis, we adopted the PM1 evidence clause accordingly [22]. Furthermore, while the c.1496 + 1G > T variant does not lead to premature stop codon formation, which might typically induce nonsense-mediated mRNA decay (NMD), we opted to directly apply the PVS1 classification based on the ClinGen SVI splicing subgroup’s guidance [23], owing to the disruption of the essential SWIRM domain. Therefore, the c.1496 + 1G > T variant was classified as pathogenic.

## Discussion

Herein, we described a 24-year-old proband with moderate DD/ID, language delay, cryptorchidism, hypotonia, behavioral abnormalities, sexual dysfunction, dense hair, craniofacial anomalies, allergic purpura and eczema, and drug allergies caused by a novel *de novo* splicing variant, c.1496 + 1G > T, in the *SMARCC2* gene. His twin brother had the same genotype and most of the phenotypes were consistent. Transcriptional level analysis based on patient-derived samples showed that this canonical splicing variant causes two forms of splicing abnormalities: in-frame insertion of partial intron 16 or in-frame deletion of exon 16, which may escape NMD; this has already been observed in another splice donor variant, c.1833 + 1G > T [6]. Further structural analysis showed that in-frame changes caused by the c.1496 + 1G > T variant affected the assembly of the α-helices of the SMARCC2 protein. To the best of our knowledge, this is the first *SMARCC2* mutation patient to seek medical assistance owing to reproductive needs. After a joint evaluation by a Reproductive Medicine and Genetics specialist, the proband was recommended third-generation in vitro fertilization (IVF) treatment.

The SMARCC2 protein contains five well-defined structural domains that are sequentially arranged from the N- to C-terminus as MarR-like (10–136), BRCT (140–183), Chromo (189–217), SWIRM (424–521), and SANT (596–647) domains (https://www.uniprot.org/uniprotkb/Q8TAQ2). The SWIRM domain mainly mediates specific protein-protein interactions [1, 24, 25], whereas the SANT domain is a chromatin-binding region of the protein that is thought to interact with unmodified histone tails [26]. In addition to the variants presented in this study, 45 NDD-related *SMARCC2* variants have been reported to date, including 26 missense/in-frame and 29 truncating variants (e.g., nonsense/frameshift variants and splicing variants that result in out-of-frame changes of the protein). In addition to the variants reported in this study, four variants (c.1311-1G > A, c.1311–3 C > G, c.1496 + 1G > T, and p.Arg443Trp) in the SWIRM domain have been documented, and only c.1311–3 C > G was confirmed to cause NMD. Interestingly, missense and in-frame variants frequently occur in the SANT domain [16]. Notably, there were distinct phenotypic differences between the two patient groups. Among the patients with truncating variants, approximately 70% developed DD/ID, with the majority (76%) presenting with mild cases. In contrast, the missense/in-frame variant group had 100% incidence of DD/ID, with most cases being moderate-to-severe (81%). Additionally, patients with missense/in-frame variants had a higher incidence of hypotonia [16]. These results suggested that the *SMARCC2* variant may not contribute solely to the disease via haploinsufficiency. For missense/in-frame variants, there may be additional pathogenic mechanisms, such as dominant-negative effects [16]. Although c.1496 + 1G > T is located in the SWIRM domain, our patient had moderate DD/ID and hypotonia, which further supports the conclusion that missense/in-frame variants of *SMARCC2* are associated with a more severe neurological phenotype.

Bosch et al. summarized the clinical characteristics of 65 patients and found that it may be inappropriate to classify SMARCC2-related disease as CSS, particularly due to the absence of some representative features, such as hypoplasia of the finger/toenails or the absence of the distal phalanx of the fifth digit and prominent interphalangeal joints. These features were not observed in twin patients. Furthermore, the facial malformations in patients with *SMARCC2* variants lack specificity. Among the 11 reported features, abnormalities in the external ear and thin upper lip vermilion occurred with a frequency exceeding 40% [16]. In our patients, in addition to the previously reported facial features of a prominent forehead, wide nose, low-set ears, and thin upper lip vermilion, we also discovered features of the epicanthus, which have rarely been described previously (Table 1). Therefore, we agree and recommend naming SMARCC2-related diseases separately and distinguishing them from CSS.

The phenotypes of most of our patients were consistent with those of previously reported patients, including core neurological features. However, we still found some previously unreported features, such as sexual dysfunction, allergic purpura, and drug allergies (Table 1). We particularly noticed the clinical features of erectile dysfunction in these twin patients because it may be an easily overlooked phenotype. In contrast, 12 of the 65 patients with *SMARCC2* variants were adults, indicating that their assessment should be combined with developmental characteristics. This is particularly important because for most rare genetic diseases, we may not have the opportunity to collect data from adult patients; therefore, we generally focus on describing the medical history of pediatric patients. This may have caused us to miss the optimal assessment time, resulting in new risks for adult patients. A recent study of 35 adult patients showed that being overweight and obese were common in adult patients with CSS. In addition, the incidence of visual impairment, scoliosis, and behavioral abnormalities is significantly higher in adults [27]. Therefore, it is necessary to continuously follow-up the *SMARCC2* mutant cohort, describe the clinical characteristics of all age groups, and draw a natural history of the disease.

In summary, we herein described a pair of twin adult patients harboring a novel splicing variant in the SWIRM domain of SMARCC2, which resulted in in-frame changes of the protein. Our findings reinforce the link between non-null variants of *SMARCC2* and more severe neurological phenotypes, and underscore the need for distinct disease nomenclature in such cases. This study also highlights the importance of breaking away from the phenotypic framework of pediatric patients to conduct a comprehensive phenotypic assessment of adult patients harboring *SMARCC2* variants.

## Data Availability

The raw data of WES for this article is not publicly available because of privacy concerns from the family. Requests to access these datasets should be directed to the corresponding authors.

## Abbreviations

BAF: BRG1-associated factor
CNV: Copy number variation
CSS: Coffin-Siris syndrome
DD: Developmental delay
ID: Intellectual disability
IVF: In vitro fertilization
NDD: Neurodevelopmental disorder
NMD: Nonsense-mediated mRNA decay
PBMC: Peripheral blood mononuclear cell
RT-PCR: Reverse transcription PCR
WES: Whole-exome sequencing
WT: Wild-type
